# Dietary Habits of Pregnant Women in Spain: The Role of Nutrition Education in Midwife Consultations

**DOI:** 10.3390/nu17010120

**Published:** 2024-12-30

**Authors:** M. Josefa Olloqui-Mundet, Marta Palma-Morales, M. Carmen Cantarell-González, M. Mar Cavia, Sara R. Alonso-Torre, Olga Ocón-Hernández, Celia Rodríguez-Pérez, Celia Carrillo

**Affiliations:** 1 Área de Nutrición y Bromatología, Facultad de Ciencias, Universidad de Burgos, Plaza Misael Bañuelos s/n, 09001 Burgos, Spainmmcavia@ubu.es (M.M.C.); salonso@ubu.es (S.R.A.-T.); 2 Departamento de Nutrición y Bromatología, Facultad de Farmacia, Universidad de Granada, Campus de Cartuja, s/n, 18071 Granada, Spain; martapalm@ugr.es; 3 Instituto de Nutrición y Tecnología de los Alimentos (INYTA) ‘José Mataix’, Centro de Investigación Biomédica, Universidad de Granada, 28100 Granada, Spain; 4 Unidad de Ginecología y Obstetricia, Hospital Universitario San Cecilio, 18007 Granada, Spainooconh@ugr.es (O.O.-H.); 5 Instituto de Investigación Biosanitaria ibs.GRANADA, 18016 Granada, Spain

**Keywords:** pregnancy, dietary habits, nutritional education, knowledge, midwife

## Abstract

**Background & Objectives:** Correct nutrition during pregnancy is key to guaranteeing success at this stage of a woman’s life, and nutritional education is the fundamental tool for achieving this. Studies carried out in different countries indicate that pregnant women do not comply with dietary and nutritional recommendations. Given the lack of evidence available in Spain and the importance of this knowledge to be able to assess the need for nutritional intervention in this group, the aim of this study focused on the current status of the issue in Spain: the quality of the diet of Spanish pregnant women and its conditioning factors. **Methods:** Two representative regions of the country were selected, one located in the north of Spain (Burgos) and the other in the south (Granada), and a descriptive, cross-sectional observational study (sample size: 771) was carried out using a questionnaire administered at the University Hospital of Burgos and the Hospital Clínico San Cecilio in Granada, which had previously been subjected to a process of evaluation by expert judgement. **Results:** Pregnant women presented an adequate diet quality (8.0 ± 2.0), according to the questionnaire used, despite their poor knowledge of food and nutrition (4.9 ± 1.6 out of 10). However, deficiencies were detected in the consumption of very interesting food groups from a nutritional point of view, such as legumes, nuts and fish (just 29.4%, 37.6% and 24.8% of the pregnant women met the recommendations, respectively) and insufficient physical exercise. The eating habits of pregnant women depend on their age, their country of origin, their level of education, their pre-pregnancy BMI, the knowledge acquired during pregnancy and the degree to which they put into practice the advice received from their midwife. Most pregnant women do not change their habits during pregnancy, although there are positive trends in this respect. **Conclusion:** The quality of the diet of the Spanish pregnant women surveyed, and their level of physical activity, could be improved by enhancing the nutritional education they receive during this stage of life. The role of the dietician in this respect, as part of multidisciplinary teams, should be the basis for future research.

## 1. Introduction

Pregnancy is a time in a woman’s life when a series of physiological changes occur in order to achieve optimal fetal growth and development, as well as prepare the mother’s body for breastfeeding. To cope with these changes, energy and nutrient requirements increase during pregnancy [1].

Thus, a healthy diet, based mainly on plant-based foods, as is the case of the Mediterranean dietary pattern, is essential to meet the requirements and, thus, engsure the health of the mother and the proper development of the fetus.

Several studies agree that pregnant women do not comply with the recommendations regarding food and nutrition [2,3]. In fact, several authors have found low adherence to the Mediterranean dietary pattern in pregnant women from different Mediterranean countries [4,5,6] despite the benefits associated with the consumption of this diet during pregnancy [7,8]. Nutrition education during pregnancy can improve the knowledge and habits of pregnant women [9,10]. In this regard, it is noteworthy that, surprisingly, not all pregnant women receive nutritional education during this stage of life [11,12,13] despite the recommendations of the World Health Organization (WHO) in this regard [14]. Among the women who do receive it, some consider that they have received it too late, while others find it too general, unspecific, insufficient, impractical or even inadequate, as it omits many relevant issues [11,15,16,17,18,19,20,21]. This poor, limited and sometimes contradictory nutritional education [11,12,15,22] may be behind the lack of food and nutrition knowledge observed among pregnant women [3,21].

However, it should be noted that the available studies in this regard are limited to certain geographical origins [23]. Although the dietary habits of pregnant women have been extensively studied in different countries [24,25,26,27,28,29], the relationship between these habits and the nutritional education received during pregnancy has been mainly addressed in Australia, the USA, Brazil, Asian countries and Africa. In Europe, the available studies are limited to France [30], Norway [15], United Kingdom [31], the Czech Republic [21] and Romania [32]. Considering that there is great heterogeneity in relation to those responsible for nutrition education of pregnant women, and/or their competencies, at the international level, the extrapolation of results between countries is complicated.

Thus, this study aims to determine the current status of the subject (the dietary habits of pregnant women, their knowledge of food and the nutritional education received) in Spain, as no similar study has been carried out in this country to date. Bearing in mind that, at present, one of the central figures in the nutritional education of pregnant women in the Spanish public health system is the midwife (health education is one of the criteria proposed by the current legislation to carry out pregnancy control and monitoring by midwives), this study would allow, further than assessing the need for nutritional intervention in Spanish pregnant women, to highlight, if necessary, the importance of a multidisciplinary team (including the dieticians) in the nutrition education of key physiological stages such as pregnancy.

## 2. Materials and Methods

### 2.1. Study Design, Population and Sample Size

A cross-sectional descriptive observational study was carried out to determine the habits, knowledge and nutritional education received by Spanish pregnant women. For this purpose, two Spanish regions were selected, one in the north (Burgos) and the other in the south (Granada) with their own climatic, cultural and socioeconomic characteristics, and, therefore, representative of the diversity of the Spanish territory.

Data were collected by means of a self-administered questionnaire at the Hospital Universitario de Burgos (HUBU) (public hospital in Burgos, Castile and Leon) and at the Hospital Universitario Clínico San Cecilio de Granada (HUSC) (public hospital in Granada, Andalusia).

Women were included in the study who were at 38–42 weeks of gestation and who attended the HUBU or the HUSC for their last pregnancy follow-up visit. Pregnant minors and those who did not have a minimum knowledge of Spanish to be able to adequately complete the questionnaire were excluded from the study.

The sample size was estimated using the calculator provided by Fisterra [33] taking into account the number of births registered in 2020 (1502 in HUBU; 2800 in HUSC), a confidence level of 95%, a precision of 5% and a proportion of 50%. The estimated sample size was 306 in Burgos and 338 in Granada (360 and 398, respectively, considering a sparse proportion of losses of 15%).

### 2.2. Questionnaire Design

The questionnaire was designed on the basis of previous work [34] and making the appropriate adaptations after defining the construct of the present study. Once the complete questionnaire had been designed, it was subjected to an expert judgement evaluation [35]. This assessment was carried out by researchers with extensive experience in the field of survey validation, as well as by two experts in the field of food and nutrition. Thus, a total of si experts completed this stage of the process, thus fitting within the recommended range of evaluators for the type of assessment [36].

To carry out the assessment, each expert had to independently rate each question or item of the questionnaire according to four categories: sufficiency, clarity, coherence and relevance [35]. All categories were defined on a scale of 1 to 4, where 1 and 4 referred to the lowest and highest level of compliance, respectively. In addition, there was a comment section for each item, so that experts could make any comments they considered appropriate. The scores given by each of the experts were combined and the percentage of validity was calculated for each item. The acceptable level was set at 80%, as this is the minimum value considered to be compatible with adequate validity [36]. Thus, the questionnaire, which initially consisted of 62 questions, was reduced to a total of 58 items after the expert judgement process. Five questions were eliminated (three for not reaching the minimum score in the category of coherence and relevance and two after discussion with the experts), one new question was added, four questions were reworded and six were moved. Finally, the questionnaire was tested on a sample of 10 pregnant women.

### 2.3. Structure of the Questionnaire

The questionnaire (available on request) was structured in five sections:

Section 1 (eight questions). Socio-demographic questions and characteristics of the participants.

Section 2 (11 questions). Nutritional education received during pregnancy. Information on the type and frequency of information received was collected through closed-ended questions. Likert-type questions (with a 5-point scale, where 1 indicated “not at all” and 5 indicated “very much”) were used to find out the depth with which different topics had been covered, and their interest or satisfaction in this regard, among other issues related to the nutrition education received.

Section 3 (six questions). Weight monitoring during pregnancy. Information was collected on issues related to the pregnant woman’s weight, its monitoring by the midwife and her perception of it, by means of closed-ended questions; in two cases, 5-point Likert-type questions were used.

Section 4 (17 questions). Pregnant women’s dietary habits. The quality of dietary habits was estimated using the MEDAS-14 questionnaire [37], a validated tool that determines adherence to a Mediterranean diet, on the understanding that this dietary pattern is a reference point for dietary quality. Gesteiro et al. [38] adapted this questionnaire (originally consisting of 14 items) for the gestational period (excluding wine in the final calculation, since, although it is considered a component of the Mediterranean diet, its consumption is totally discouraged during pregnancy). Compliance with the intake recommendations for each item counts for 1 point. The quantitative variable “adherence to the Mediterranean diet” was categorized into two levels: “Low adherence” (less than 7 points) and “High adherence” to the Mediterranean diet (equal to or greater than 7 points), applying the cut-off points suggested by Gesteiro et al. [38]. A question on physical exercise was also included in this block. The quality of the self-referred diet was assessed by means of a 5-point Likert-type question. In order to estimate changes in dietary habits (or physical activity) during pregnancy, each question of the MEDAS-14 questionnaire was followed by a sub-question aimed at assessing whether the consumption of that food had increased or decreased compared to the pre-pregnancy stage or whether, on the contrary, it had remained at similar levels. Information was also collected on the supplements consumed by pregnant women during pregnancy.

Section 5 (16 questions). Knowledge of food and nutrition in pregnant women. A total of 10 closed questions were included, four with a single answer and six with multiple answers. In all cases, a “don’t know/no answer” option was left to avoid forcing the answer and that part of the correct answers were the result of chance. To obtain the total score for the knowledge test, each of the single-answer questions was given a score of 1g point (4 points in total). Within the multiple-choice questions, 1 point was awarded for each correct answer (even if not all of the correct options in the question were marked), so that this block of questions was assessed out of a total of 22 possible points. Taking these assumptions into account, the maximum score for the knowledge test was set at 26 points, although the final mark was expressed out of 10.

Questions were also added to find out the pregnant women’s perception of the importance of good habits during pregnancy or their level of self-reported knowledge (using 5-point Likert-type questions), among others.

### 2.4. Dissemination of the Questionnaire

The questionnaire was administered in paper format during the last pregnancy follow-up visit, which takes place at approximately week 40. Pregnant women completed the survey during the fetal monitoring test (under the guidance of a researcher who provided the necessary clarifications for the correct completion of the questionnaire), within the HUBU or HUSC facilities. The data collection period was carried out during the years 2021–2022.

### 2.5. Data Analysis

Statistical analysis of the data was carried out with Statgraphics 19. A descriptive analysis of the data was performed using means and standard deviations (in the case of quantitative variables) or frequencies and percentages (in the case of qualitative variables). Some quantitative variables, such as age or BMI, were categorized. The five-category Likert-type scales were reduced to three levels: “low” (scores 1–2), “moderate” (score 3) and “high” (scores 4–5), to simplify the analysis. The Chi-square test of independence was used to determine associations between qualitative variables (*p* < 0.05 was set as the level of statistical significance). The Kolmogorov–Smirnov test was used to assess the distribution of variables. Comparisons of independent means were performed using Student’s *t*-test. Pearson’s correlation test was used to test correlations between variables.

### 2.6. Ethical Considerations

The bioethics committee of the University of Burgos (IR4/2019), the University Hospital of Burgos (CEIC 2184) and the Bioethics Committee of Andalusia (IR 4/2019) approved the research study. Informed consent was included in the first part of the questionnaire. This provided a brief explanation of the study’s objectives, allowing participants to make an informed and voluntary decision regarding their participation. All data were collected in a completely anonymous and confidential manner.

## 3. Results

### 3.1. Descriptive of the Sample

A total of 789 questionnaires were collected, 397 in Burgos and 392 in Granada. After an initial review, 18 were eliminated because they were not correctly completed. Thus, we worked with data from 771 pregnant women.

Table 1 shows the main characteristics of the respondents.

The mean age of the Spanish pregnant women surveyed was 33.4 ± 5 years. Ninety percent of the participants were of Spanish nationality and the remaining percentage were foreign nationals. Of the sample, 39.3% reported having a university education. Almost half of the pregnant women (49.8%) reported no previous pregnancies at term, 35.6% reported one previous pregnancy and 14.6% reported two or more.

A total of 35.4% of the participants said that they had had an abortion in the past, while 87.9% of the pregnant women reported not having suffered from any pre-pregnancy illnesses, although the remaining 12.1% confirmed different pathologies such as obesity or diabetes, among others. The majority of those surveyed (81.3%) stated that they had no health problems during pregnancy; among the 144 pregnant women who experienced some alteration or pathology derived from pregnancy, the most common were gestational diabetes, blood pressure alterations (hypertension and pre-eclampsia), hypothyroidism and COVID-19, in order of frequency. In view of these data, it can be affirmed that most of the women in the study sample faced gestation without underlying pathology and enjoyed their pregnancy without notable complications.

The mean pregestational BMI of the participants was 24.3 ± 5.1 kg/m^2^. The majority (61.8%) of the respondents faced pregnancy at a normal weight, 20.6% were overweight, 12.9% were obese and a low percentage (4.7%) were underweight before pregnancy. Thus, 38.2% of the women began pregnancy with a weight far from what is considered healthy. Specifically, 33.5% were above the recommended weight for their height.

No significant differences were observed between pregnant women from Burgos and Granada in relation to the characteristics described above, with the exception of country of origin, while the percentage of non-Spanish pregnant women was higher in the sample from Burgos. Hispanic Americans were the most represented in both samples.

### 3.2. Dietary Habits During Pregnancy

The mean adherence of the participants to the Mediterranean diet was 8.0 ± 2.0. According to the categories defined in this study, the majority of pregnant women (77.8%) showed good adherence to the Mediterranean dietary pattern.

It is worth noting that when the pregnant women were asked about the quality of their diet during pregnancy, 60.7% considered it to be very adequate. Furthermore, the results indicated that the quality of their self-reported diet was related (*p* < 0.05) to the quality of their diet as measured by the degree of adherence to the Mediterranean pattern. Thus, the percentage of pregnant women who considered their dietary habits to be very good during pregnancy was higher among those who showed greater adherence to the Mediterranean pattern (67.3% and 37.6%, for high and low adherence to the Mediterranean pattern, respectively).

Table 2 shows the pregnant women’s compliance with each of the items included in the MEDAS-14 questionnaire, as well as their compliance with the physical exercise recommendations.

Most pregnant women (95.6%) confirmed using olive oil as their culinary fat of choice; 40.9% of the pregnant women consumed four tablespoons or more of olive oil per day, thus meeting the recommended amounts. A total of 57.3% of the pregnant women consumed two or more servings of vegetables daily, thus meeting the recommended amounts for this food group. As in the case of vegetables, slightly more than half of the sample (50.3%) complied with the recommended consumption of three or more pieces of fruit per day. The majority of women (80.8%) reported an intake of less than one portion of red meat, sausages, hamburgers or processed meat per day, thus complying with the recommendations. On the other hand, the majority of respondents (91.6%) complied with the recommendation to eat less than one daily serving of butter, cream or margarine and less than one carbonated and/or sugared drink per day (89.5%). Only 29.4% reported a consumption of three or more servings of pulses per week. Similarly, in the case of fish and seafood consumption, 75.2% of the sample confirmed a lower-than-recommended consumption, with less than three servings per week of this food group. A total of 42.7% of the respondents showed an excess consumption of commercial confectionery, with an intake of 2 portions or more per week. Most of the pregnant women did not comply with the recommendations for nut consumption, with only 37.6% confirming an intake of more than three servings per week. A total of 86.5% reported choosing turkey, chicken or rabbit meat instead of pork, beef, hamburgers or sausages, thus complying with the recommendations. With regard to “sofrito”, which is a typical Mediterranean sauce made with a base of tomato, onion or leek, garlic and olive oil, almost three quarters of pregnant women (61.7%) reported consuming it two or more times a week to dress rice, pasta, vegetable or other dishes, thus conforming to the characteristics of Mediterranean cuisine.

It is worth noting that the percentage of pregnant women who complied with the recommendations for the consumption of olive oil, nuts, red meat, pastries and “sofrito” was higher among women from Granada. In contrast, pregnant women from Burgos were more in line with the recommendations for fruit, fish and carbonated and/or sugary drinks.

Almost all pregnant women (92.1%) reported never drinking wine, although 7.9% reported an intake of less than seven glasses per week.

The majority (81.6%) of respondents took folic acid supplementation during pregnancy, although only 16.5% combined it with iodine. In terms of supplementation, 19.6% of the pregnant women reported taking multivitamins.

Finally, in relation to physical exercise, only 45.9% of the Spanish pregnant women surveyed confirmed that they comply with the World Health Organization’s recommendations of 30–45 min of moderate physical activity per day [23], which means that more than half, 54.1%, move less than recommended or do not do any physical exercise at all.

When evaluating the association between the different socio-demographic variables (and characteristics of the pregnant women) and their dietary habits (Table 1), it was observed that the dietary habits of the pregnant women in the study were related to their age, country of origin, educational background and pre-pregnancy BMI (*p* < 0.05). The percentage of pregnant women with a low adherence to the Mediterranean diet was higher among younger women (under 35 years of age) and those with less education. Similarly, the percentage of pregnant women who began pregnancy with obesity was higher among those with low adherence to the Mediterranean pattern. Among non-Spanish pregnant women, there was a higher percentage of women with low adherence.

In contrast, the dietary habits of the respondents were found to be independent of their underlying pathologies, previous pregnancies or miscarriages and quality of gestation.

Although there is some difference in the significance of the variables–habits associations when the analysis is carried out in each sample (Burgos and Granada) independently, the trends described for the total sample are similar in pregnant women from both origins.

It should be noted that physical activity is related to the degree of adherence to the Mediterranean pattern (*p* < 0.05). Thus, the percentage of pregnant women who comply with the physical activity recommendations is higher among those with better dietary habits (49.5% and 35.1%, for high and low adherence to the Mediterranean pattern, respectively) and vice versa (64.9% of pregnant women with low adherence to the Mediterranean pattern do not comply with the minimum level of daily movement, compared to 51% who do not within the group of pregnant women with better habits).

### 3.3. Influence of the Gestational Period on Dietary Pattern

Table 3 shows changes in the intake of different foods during pregnancy. Changes in physical exercise are also indicated. As detailed in the Material and Methods Section, each of the 14 questions comprising the MEDAS-14 questionnaire was completed with a sub-question (“Have you changed your intake of … during pregnancy?”) in order to determine whether the pattern of consumption of each food group (or physical exercise) was similar in the pre-pregnancy period or whether it had changed (increased or decreased) during pregnancy.

Most of the Spanish pregnant women surveyed did not modify their intake of foods characteristic of the Mediterranean pattern such as olive oil (84.5%), vegetables (60.9%), pulses (77%), fish/seafood (74.9%), nuts (67.3%) or “sofrito” (79.2%) during the gestational period. However, it is worth noting that among those pregnant women who did modify their intake of these food groups, the women who increased their consumption during pregnancy were more likely to do so, except in the case of olive oil and “sofrito”, where most pregnant women reported lower intakes during pregnancy. In the case of fruit, almost half of the pregnant women (48.8%) reported having increased their intake as a result of pregnancy.

As mentioned above for typical Mediterranean foods, the majority of pregnant women did not change either their consumption of non-Mediterranean foods (red meat, butter/cream, sugary drinks and commercial confectionery) during pregnancy. However, among the pregnant women who did modify their intake of these foods, a trend opposite to that described for Mediterranean foods was observed, i.e., the percentage of women who reduced their consumption of less healthy foods during pregnancy predominated, with the exception of commercial confectionery, where 23.7% of the sample claimed to have increased their consumption during this period.

As far as wine is concerned, practically half of the pregnant women (48.6%) confirmed a decrease in wine intake during the gestational period and the remaining half stated that they had maintained a similar intake during this stage, all of them coinciding with women who were abstainers in the pre-pregnancy stage. Surprisingly, two women stated that they had increased their wine consumption during pregnancy, although we cannot rule out the possibility that these responses may be due to a recording error or a misunderstanding of the item by the respondent.

In the case of physical activity, 18.9% of the women surveyed increased their level of daily movement during pregnancy, compared to 42.2% who said that they had not changed their activity during this stage. A significant percentage of the sample (38.9%) reported having reduced their physical activity since the beginning of pregnancy.

No significant differences were observed between pregnant women from Burgos and those from Granada in relation to changes in habits associated with pregnancy, except in the case of red meat, where pregnant women from Granada showed slightly more positive changes in the consumption of this food group.

Table 3 also shows the changes in the consumption of different foods grouped according to the quality of the pregnant woman’s diet (low or high adherence to the Mediterranean diet).

There is an association between the quality of the respondent’s diet and changes in the consumption of vegetables, fruit, red meat, nuts and commercial confectionery. Thus, the percentage of pregnant women who increase their intake of vegetables, fruit and nuts is higher among those with a higher quality diet and the opposite trend is observed in women who decrease their consumption. In the case of red meat and commercial bakery products, the percentage of pregnant women who reduce their intake is higher among those with better dietary habits and the opposite is true for women who increase their consumption. No association was observed between the quality of a woman’s diet during pregnancy and changes in the consumption of the other foods analyzed.

It should be noted that when the associations between diet quality and changes in consumption habits were studied independently for the Burgos and Granada samples, some of the aforementioned significance changed, although the trends observed confirm the previous findings, with a higher percentage of pregnant women increasing their consumption of healthy foods and decreasing their consumption of less healthy foods.

The change in physical exercise during pregnancy is independent of the quality of the respondents’ diet (*p* > 0.05).

### 3.4. Knowledge of Food and Nutrition in Pregnant Women

Table 4 shows the distribution of correct and incorrect responses to the questions on food and nutrition knowledge asked to pregnant women.

Within the block of single-answer questions, the one with the highest percentage of correct answers was the question asking if there are any differences in energy needs during the different trimesters of pregnancy. Eighty-nine percent of the respondents stated that they knew there were differences in the needs related to the different stages of pregnancy. Most pregnant women (63.8%) were aware of the recommendations for dairy intake during pregnancy, although it should be noted that there is a high level of ignorance in this regard (36.2%). The questions with the lowest percentage of correct answers were related to recommendations for folic acid intake and weight gain. Almost half (49.3%) of the pregnant women wrongly considered that folic acid supplements should be consumed throughout pregnancy, and only 33% were aware of the actual recommendations for folic acid supplementation (at least one month before pregnancy and during the first three months of pregnancy). Only 22% of respondents were aware of the recommended range of weight gain for people starting pregnancy with a BMI in the normal weight range (18.5–24.9 kg/m^2^) and most considered the appropriate gain to be between 9–12 kg.

In relation to the multiple-choice questions, the question corresponding to the recommended guidelines to combat constipation received the highest percentage of correct answers, with 62.1% of the pregnant women able to identify the three correct guidelines (adequate intake of water, physical activity and fruit and vegetables) within the proposed list. The rest of the questions obtained a very low percentage of correct answers.

Specifically, only 18.3% of the respondents identified all foods that should be avoided due to the risk of listeriosis or toxoplasmosis, although it should be noted that the majority of pregnant women were aware of the importance of avoiding cured meat products that had not undergone heat treatment, such as chorizo, salami or cured ham (93.8%), raw fish or seafood (85.5%), unpasteurized milk and milk products (82%), non-sterilized refrigerated pâtés (79.6%) or smoked fish products requiring refrigeration (62.3%). However, only 23.2% were able to identify bagged salads as potential vehicles for Listeria monocytogenes poisoning.

Only 7.3% recognized iron-rich foods in the proposed list. Legumes were identified as a source of iron by a large majority of respondents (86.5%), followed by green leafy vegetables (50.1%). Surprisingly, less than half of the pregnant women identified red meat as an iron-rich food (47.7%) and a minority (20.8%) identified fish within this food group. Even lower (3.1%) was the percentage of pregnant women who knew which foods had higher quality iron (meat and fish, as opposed to those of vegetable origin). In this regard, it should be noted that legumes were, erroneously, the choice of most of the respondents (51.8%), well above red meat.

Only 4.3% of pregnant women correctly identified all the guidelines aimed at reducing episodes of nausea and vomiting, which are common in pregnancy. Although the majority of respondents (68%) recognized that avoiding fatty or spicy foods can help with these episodes, less than half (39.8%) identified eating something solid in the morning as an appropriate guideline and only 15.6% highlighted the minimization of odors in these cases.

Finally, only 2% of pregnant women recognized the four fish that should be avoided during pregnancy because of their high mercury content. Although the majority of respondents identified swordfish (66.7%), bluefin tuna (64.9%) and shark (55%) in this respect, only 9.8% recognized pike as a potential source of heavy metals. Notably, 55% of pregnant women wrongly included tuna in this group of fish to avoid.

The mean score obtained by the pregnant women in the knowledge test did not exceed 5 points out of a total of 10 (4.91 ± 1.55). No significant differences were observed between the knowledge of the pregnant women from Burgos and Granada, although it should be noted that the percentage of pregnant women who knew the recommendations for dairy products and fish to avoid during pregnancy was higher among those from Burgos. Significant differences (*p* < 0.05) were observed in the scores obtained according to the dietary habits of the Spanish pregnant women, with greater knowledge among those who showed better diet quality (5.36 ± 1.68 and 4.57 ± 1.38, for high and low adherence, respectively); and with marked differences in the case of pregnant women from Granada (7.57 ± 0.43 vs. 4.61 ± 1.28, for high and low adherence, respectively). The correlation between both continuous variables was significant (*p* < 0.001).

Before objectively assessing the pregnant women’s knowledge of food and nutrition, one of the questions in the questionnaire asked them to assess their knowledge of food and nutrition before they became pregnant. In this regard, half of the respondents (50.8%) said that they had a lot of knowledge on the subject and only a minority (9.9%) considered that they had a low level of knowledge. The results indicated that the quality of the diet of pregnant women is related (*p* < 0.05) to this self-reported knowledge, so that the percentage of women who considered having a lot of knowledge in this field was higher among those who showed better habits during pregnancy (55.4% and 34.3%, for women with better and worse dietary quality, respectively) and vice versa (20.1% of pregnant women with worse habits admitted to having low dietary knowledge, compared to 7% of those who admitted the same in the group with greater adherence to the Mediterranean pattern). Similar trends between samples (Burgos and Granada) were found when the data were analyzed independently for each sample.

In addition to measuring knowledge objectively, and as detailed above, we also assessed the pregnant woman’s perception of the importance of correct eating habits during pregnancy. For this purpose, three questions were used, which together with their corresponding answers are shown in Table 5. A large majority of the respondents are aware of the importance of nutrition during this stage of a woman’s life cycle (95.7%); of the risks of inadequate weight gain during pregnancy, both for the mother’s health and that of the child (86.4%); and of the great repercussions that eating habits can have on the baby’s health (93.7%). The results indicate that the quality of a pregnant woman’s diet is related to the latter two issues. In this sense, the percentage of women who consider inadequate weight gain as a high risk for the health of mother and baby is higher among those who follow a higher quality diet (88.6% and 78.8% for good and low adherence to the Mediterranean pattern, respectively). Similarly, 95.3% of pregnant women with better dietary habits consider that the impact of diet during pregnancy on the baby’s health is high, compared to 88.2% of pregnant women with poorer habits. Similar trends between samples (Burgos and Granada) were found when the data were analyzed independently for each sample.

### 3.5. Nutrition Education During Pregnancy

Table 6 presents different issues related to the nutritional education received by the pregnant woman in the midwife’s consultation.

A total of 20.1–31.9% of the sample analyzed stated that they did not receive information (orally or in written material, respectively) on food and nutrition during their pregnancy follow up sessions.

Of those pregnant women who did receive information, 24.6% reported receiving little information on food and nutrition and 36.2% considered the amount to be moderate. Half (51.2%) received such information at the first pregnancy follow-up visit and only 17.1% reported receiving information on food at each follow-up visit. In terms of the type of information, most participants reported receiving general information. Only 15.8% of respondents highlighted personalized guidelines. When asked to indicate, from a list of 10 topics (“general advice on nutrition in pregnancy”, “influence of diet on foetal development”, “risk of over/underweight in pregnancy”, “diet in pregnant women with medical conditions (HTN, gestational diabetes…)”, “vegan or vegetarian diets in pregnancy”, “treatment of gastrointestinal problems (nausea, heartburn, constipation…)”, “caffeine, tobacco, alcohol and other drugs”, “nutritional supplements in pregnancy”, “food safety in pregnancy (toxoplasmosis, listeriosis…)” and “physical activity during pregnancy”), the depth with which they had been dealt with in the midwife’s consultation, “general advice on nutrition in pregnancy”, “food safety” and “physical activity in pregnancy” stood out as having been dealt with in greater depth. In contrast, issues related to supplementation in pregnancy, gastrointestinal problems, diet in pregnant women with clinical problems and vegan or vegetarian diet were covered in less depth. All topics on the list were rated as very important by more than 80% of the pregnant women, with the exception of “diet in vegan or vegetarian pregnant women”.

Although 49.5% of the respondents considered that their knowledge of nutrition had increased a lot during pregnancy, half of the respondents rated this increase as little (23.7%) to moderate (26.7%). Only 10.9% of the pregnant women said that they had had contact with a dietician during pregnancy. Regarding the usefulness of the knowledge acquired, the majority of the pregnant women (70%) confirmed that they put this knowledge into practice a lot. Those who did not follow the advice received stated that the main reason for not doing so was their preference to continue with their eating habits (44.9%).

It is worth noting that 82.7% of the Spanish pregnant women surveyed consider the information on feeding provided by the midwife to be very credible. However, although the majority of pregnant women (52.2%) confirmed a high degree of satisfaction with the information on nutrition received at the midwife’s consultation, 132 pregnant women (20.1% of the sample) admitted to being dissatisfied with the advice received in this regard.

A total of 87.4% of the pregnant women said that the midwife had monitored their weight gain during pregnancy and that they had been informed about it. One in 10 pregnant women felt uncomfortable with such monitoring.

The percentage of pregnant women who received information or consultation material on dietary issues in the midwife’s consultation was higher (*p* < 0.05) in the Burgos sample than in the Granada sample. In fact, a greater number of pregnant women from Burgos quantified the amount of information received as “a lot”, and rated their satisfaction with it as higher. Despite this, no differences were observed between Granada and Burgos in the self-perceived increase in knowledge.

When analyzing the relationship between the nutritional education received and the habits of the pregnant women (Table 6), it was observed that the quality of the diet of the respondents was not associated with the fact of having received information on food and nutrition (neither verbal nor written). Similarly, the habits of the respondents did not depend on the type of information received (personalized/general information) nor on the quantity; nor did they depend on the satisfaction of the pregnant woman with the information.

On the contrary, the habits of pregnant women differed between those who followed the recommendations received and those who did not. The percentage of pregnant women who most obeyed the dietary advice received was higher among women with good adherence to the Mediterranean diet (73.7%, compared to 57.4% of those who followed the advice in the group with poorer diet quality). In addition, the quality of the diet of the participants was related to the knowledge acquired during pregnancy. A total of 51.9% of pregnant women with good adherence to the Mediterranean pattern confirmed that they had greatly increased their knowledge of food and nutrition during pregnancy, compared to 41.2% who said the same in the group of pregnant women with poorer dietary habits. Although in the case of the Granada sample, no significant relationships were observed, the trends in this group are similar to those described for the total sample.

## 4. Discussion

A diet based on the Mediterranean pattern during pregnancy is associated with multiple benefits for the mother and fetus [39,40,41,42,43,44]. For this reason, studying the dietary habits of pregnant women is a key step in detecting deficiencies and planning, if necessary, intervention strategies aimed at improving them. In this regard, this study indicates that more than three quarters of the Spanish pregnant women surveyed showed good adherence to the Mediterranean pattern during pregnancy, with a mean value of eight out of 13. These results are in line with those obtained by some authors [25,45,46,47] who also found a majority of pregnant women with good adherence to the Mediterranean pattern, but disagree with others [6,26,27]. Although the variation between the results obtained in this study and those provided by other authors could be related to differences in the dietary habits of each region, taking into account the great diversity of tools used to determine adherence to the Mediterranean diet (and cut-off points for each of them), everything would point to a methodological issue, as has been discussed in previous studies [40].

Regarding the conditioning factors of the dietary pattern of Spanish pregnant women, it was observed that the degree of adherence to the Mediterranean dietary pattern was related to the age of the pregnant women, country of origin, level of education and BMI prior to pregnancy. Consistent with the results of this study, previous research [24,48] also found that older age had a protective effect by reducing the likelihood of low adherence to the Mediterranean diet. These results could be explained by the fact that maternal age over 35 years is associated with an increased risk of complications in pregnancy [49]. Thus, it is understood that an “older” pregnant woman is likely to take more care of her habits. Several authors have also found significant influences of level of education and pregestational BMI [24,48,50]. In contrast to what was found in the sample of pregnant women studied in the present study, other authors found that the number of previous pregnancies was significantly associated with the quality of the pregnant women’s diet. In general terms, pregnant women with a lower level of education, higher pregestational BMI and previous pregnancies are more likely to have a low adherence to the Mediterranean pattern. Other factors associated with the quality of the diet of pregnant women include income level, smoking and alcohol consumption [48]. The degree of physical activity of pregnant women has also been highlighted by various authors as a factor related to diet quality [24,50], in line with the findings presented in this paper.

Regarding the frequency of consumption of the different food groups, it should be noted that almost half of the pregnant women did not comply with the recommendations for the consumption of vegetables and fruit during pregnancy and only a minority complied with the recommendations for the intake of legumes, nuts, fish and seafood. These results are in line with those obtained by other authors [27,48,51], who observed low compliance with the recommendations for consumption of these food groups during pregnancy. Thus, although the degree of adherence to the Mediterranean pattern was adequate in most pregnant women, according to the cut-off point and consequent categorization established in this study, these data should be interpreted with some caution, since only a small percentage of pregnant women complied with the recommendations for consumption of foods of nutritional interest, such as legumes, fish and nuts. It should not be forgotten that fish are a source of docosahexaenoic acid (DHA), a long-chain polyunsaturated fatty acid of the omega-3 family, whose intake during pregnancy is associated with the correct development of the retina and central nervous system of the fetus [52]. Legumes and nuts are good sources of proteins and healthy fats, respectively, and both of them are rich in fiber, which is of interest taking into account the constipation usually associated with pregnancy.

Regarding wine intake, although the majority of pregnant women did not consume wine during pregnancy, it is important to note that, although there is no evidence of a safe level of alcohol consumption during pregnancy, a small percentage of respondents do not comply with the recommendations for zero intake [53]. In this sense, the results presented are in line with those found by Izquierdo-Guerrero [54] in which 8.8% of her sample confirmed consuming alcohol (wine and beer) during pregnancy.

Less than half of the Spanish pregnant women surveyed complied with the minimum recommendations of 30 min of physical activity per day set out in most pregnancy-oriented guidelines [55]. Suarez-Martínez et al. [24] also found that most women had an inactive or sedentary lifestyle during pregnancy. Although the results observed in a prospective cohort study of 463 Andalusian pregnant women were somewhat more positive, with 54–61% of the pregnant women achieving the minimum recommended physical activity level [55], the percentage of inactivity that prevails during pregnancy is also notable. In view of these results, it could be said that practically one out of every two pregnant women in Spain does not do the minimum recommended activity. Taking into account that physical activity is beneficial for mother and child, as it is associated with good cardiovascular health, a reduced risk of chronic pathologies, a reduced risk of preterm pregnancy, and weight control during pregnancy, as well as with hypertension or gestational diabetes control [56], there is a clear need for education on the importance of an active lifestyle during pregnancy.

Pregnancy is a time when women are particularly concerned about their dietary intake and when they are most motivated to make improvements. In fact, when pregnant women are asked about the behaviors they practice to maintain a healthy pregnancy, a “healthy diet” is the most frequently mentioned [51]. To ensure the success of food and nutrition interventions during pregnancy, it is important to know the dietary changes women make when they become pregnant.

The dietary habits of the Spanish pregnant women in this study are, in general terms, similar to those of the women in the pre-pregnancy period. Among the changes observed, there is a positive trend, with an increase in the intake of healthy foods (vegetables, fruits, legumes, fish/seafood and nuts), and a reduction in the consumption of less recommended foods (red meat, butter/cream and sugary drinks). Hillier and Olander [51] conducted a systematic review of 11 papers related to behavioral changes during pregnancy and also concluded an increase in fruit and vegetable consumption. Forbes et al. [57] also confirmed this in a group of 400 Canadian pregnant women. However, despite the changes detected, a very significant percentage of the Spanish women surveyed do not reach the recommendations for consumption of these food groups, something that is also warned of in the aforementioned review [51]. In line with the results of the present study, Forbes et al. [57] also found a decrease in the consumption of meat and alcohol, as well as an increase in the consumption of sweets. The latter may be related to women’s own cravings at this stage of life [57]. In this regard, it should not be forgotten that the excessive consumption of sweets can result in an extra calorie load and inappropriate weight gain. Although there are no specific recommendations for sugar intake during pregnancy, the WHO recommends that sugars should not exceed 10% of total energy in adults [58]. Teaching pregnant women to make healthy substitutions for free sugar intake may be of interest for the design of intervention strategies in this group. These authors [51,57] also observed a reduction in the intake of “sofrito”, which could be related to the decrease in olive oil consumption detected in the present study.

This trend towards improved dietary habits did not extend to physical exercise, which deserves special attention as diet and physical activity should go hand in hand in terms of health care. Our results coincide with those observed by Román-Gálvez et al. [55] in Andalusian pregnant women, who also noted a decrease in the level of physical activity during pregnancy. Similarly, a study of Portuguese pregnant women observed a significant decrease in the level of physical activity, although this decrease was particularly associated with the abandonment of high-impact exercise [59].

Recent studies have tried to assess the reasons behind changes in habits during pregnancy. Cravings, together with concern for the baby’s health, would explain some of the observed behaviors [60]. Cravings are clear triggers for the intake of certain foods. The risks associated with the consumption of certain foods also clearly condition their consumption. Women during this stage are, therefore, exposed to contradictory internal messages that affect their intake. Health professionals should help pregnant woman to explore all these factors in order to identify practical strategies to optimize her intake [61].

A key factor to be able to initiate changes in eating behaviors is to have a good knowledge on the subject. This is the reason why the present study sought to find out what Spanish pregnant women knew about food and nutrition. The mean scores obtained in the knowledge test did not exceed a score of 5 out of 10. However, pregnant women with greater adherence to the Mediterranean pattern scored significantly higher than women with low adherence to this pattern. In fact, pregnant women with better habits passed the knowledge test, in contrast to those with poorer diet quality. However, these results should be interpreted with caution as the tool used to measure knowledge was not validated.

A significant proportion of the Spanish pregnant women surveyed were unaware of the recommendations for dairy consumption during pregnancy. The highest quality sources of iron were hardly identified by the respondents, and it was observed that, surprisingly, the majority put plant foods before animal foods in this respect, when non-heme iron is found almost exclusively in the latter. In this respect, Bookari et al. [16] also found a low level of knowledge on the subject in Australian pregnant women, highlighting the fact that 65% of their sample were not familiar with the Australian dietary guidelines. In line with our results, these authors found a low knowledge of the frequency of dairy consumption, as well as a significant percentage of pregnant women unable to recognize red meat as a source of iron.

Only one third of Spanish pregnant women were able to recognize the ideal form of folic acid supplementation. The majority of pregnant women were unable to adequately identify the range of healthy weight gain for a woman with normal pre-pregnancy weight, erroneously considering a value of 9–12 kg, probably in relation to the average value of 12 kg that is often transmitted to the population, without emphasizing that healthy weight gain depends on the baseline BMI [53] Lee et al. [12] also confirmed certain gaps in another sample of Australian pregnant women, finding a lack of knowledge similar to that observed in our study on issues related to folic acid supplementation guidelines, or the recommended weight gain for women with normal weight, among others.

There was also a certain lack of knowledge on issues related to food safety. For example, more than half of the pregnant women included tuna among the fish to avoid due to its mercury content, when the recommendations of the Spanish Agency for Food Safety and Nutrition (AESAN) make it clear that only bluefin tuna should be avoided [62]. It is important to emphasize this aspect since oily fish are an interesting source of DHA, so it is not advisable to limit consumption options. Although most pregnant women adequately identified foods that may increase the risk of listeriosis or toxoplasmosis, less than a quarter of pregnant women recognized bagged salads as a potential risk. In this regard, it is worth noting that AESAN includes prepared salads among the foods most frequently associated with outbreaks of listeriosis [63]. Other studies, on the contrary, when grouping the questions in the questionnaire according to the main topic, found the best knowledge on food safety issues. It is noteworthy that several authors have found that midwives limit nutrition education during pregnancy to emphasizing which foods should be avoided due to the risk of food poisoning, as this is the area in which they feel most confident [64].

Low levels of knowledge have also been found in pregnant women in the Czech Republic [21]. In contrast, the results obtained in a study conducted in Turkey [65], with more than 700 pregnant women, were more positive than those found in the present study, with an average score of 16 out of 25. A large majority of Turkish pregnant women correctly identified the recommended daily servings of dairy products during pregnancy, as well as the need to start consuming folic acid supplementation before the onset of pregnancy. The differences in the results found could be related to a lack of uniformity in the questionnaires used to measure knowledge, and the absence of validated tools, as well as the type of nutritional education received by pregnant women depending on the country. In this regard, two studies carried out in Spain, in 432 [54] and 103 [66] pregnant women in Madrid, also found a clear lack of knowledge on issues related to nutrition during pregnancy.

Interestingly, the self-reported knowledge of the Spanish pregnant women in the study was not in line with the test results, as the vast majority of them considered to have moderate to high knowledge on the subject. Moreover, this was confirmed by the relationship between self-reported knowledge and dietary habits. Perhaps the pregnant women were aware of issues not included in the test.

Finally, it is important to underline that, despite the low level of knowledge detected, pregnant women’s perception of the importance of good habits during pregnancy is positive. Most of the respondents are aware of the importance of good nutrition during pregnancy, as well as of the risks involved in excessive or insufficient weight gain for the mother and fetus. These results are in line with those of other studies [16,54].

The last of the objectives of this study focused on assessing the nutritional education received by Spanish women during pregnancy. Bearing in mind that at this stage, women are particularly receptive to health advice, as the future of their baby is at stake, it would be the moment to make the most of the time spent in contact with this group. In this regard, it should be highlighted that, as mentioned in the introductory section, the midwife is the health professional who monitors pregnancy in the Spanish public health system and her competencies include health education. This is the reason why all the questions in this section were aimed at finding out about the nutritional education that the pregnant woman receives at the midwife’s consultation.

The results presented indicate that although the majority of pregnant women receive information on nutrition during pregnancy, two women out of 10 said that they did not receive any information on nutrition and 24.9% of those who did receive information considered it to be insufficient. More than half of pregnant women said that their knowledge of food and nutrition had increased slightly to moderately during pregnancy. Although it could be thought that the COVID-19 pandemic and the consequent modification of the pregnancy follow-up plan (some of the visits that were carried out in person were replaced by telephone consultations) could have some impact on the results found, studies carried out before that pandemic obtained data similar to those of the present study [17]. It is also worth mentioning the fact that the vast majority of pregnant women received general information on food and nutrition, despite the effectiveness that personalized advice has shown [67]. However, in view of the results obtained, the nutritional education received by the pregnant women may have had some impact on their habits, since those who put into practice the recommendations received and those who considered that they had increased their knowledge during pregnancy had a greater adherence to the Mediterranean pattern.

In any case, despite these findings and the fact that most of the pregnant women were satisfied with the information received, as detailed throughout this study, several areas for improvement in the lifestyle of the pregnant women (diet and physical activity) have been detected, as have certain gaps in basic questions about food and nutrition. With the information available, it is difficult to assess whether the midwife provided quality nutritional education, covering all aspects of interest to this group, but this education did not sufficiently penetrate the pregnant women and, therefore, they did not put it into practice, or whether, on the contrary, the gaps and/or inadequate habits of the pregnant women are due to the fact that the nutritional education received in the midwife’s consultation has room for improvement. In this regard, studies carried out with midwives from different origins [68] show that midwives do not feel comfortable discussing food and nutrition with pregnant women. Spanish midwives considered the nutrition training they received as insufficient, which likely resulted in their poor level of knowledge and lack of confidence to give nutritional advice [69]. In this respect, it should be noted that the dietician is the most qualified professional to offer advice on food and nutrition, but the fact that the public health system in Spain does not include this figure means that this health professional is not available to the entire population. In fact, our data confirm that only 10.9% of pregnant women were in contact with a dietician during pregnancy. As several authors have shown, dietary interventions are effective [13,70,71,72,73,74,75] and the figure of the dietician increases their efficacy. Likewise, the inclusion of this professional in multidisciplinary teams increases the efficiency of the health system [76].

Pregnancy is a very interesting period to establish a change in habits, not only because of the receptiveness of women at this stage, but also because, if nutrition education is well designed, this change in habits will be sustainable over time and will ensure that the baby develops in a quality nutritional environment. Thus, nutrition education at this stage of a woman’s life cycle has a double impact, as a nutritionally well-educated child is a guarantee of a healthy adult.

## 5. Limitations of the Study

The dietary and nutritional knowledge of the pregnant women was assessed by means of a non-validated questionnaire. No validated tools have been found in the literature for this purpose, which could be the basis for future studies. We should also mention that the cross-sectional nature of the study does not allow the evaluation of longitudinal changes in dietary habits. In fact, since the completion of the questionnaire was carried out at the end of pregnancy, it is possible that there were memory biases or that the answers corresponded more to the diet in the last part of pregnancy (in case there were variations between the trimesters); in addition, we did not explain the sizes of the portions, which could translate into errors of estimation. The selection of two regions to assess habits in Spanish pregnant women could be considered a limitation of the study. However, the fact that no significant differences were detected between these regions supports the extrapolation of the data to the national level. Nevertheless, future studies including samples from different regions may be of interest to corroborate the findings obtained. The quantitative design used in this study limits the information that can be obtained from the results. A mixed approach, including structured interviews with Spanish pregnant women and a qualitative analysis of the data, could maximize the information on this group. Finally, it should not be forgotten that the COVID-19 pandemic, and the consequent restructuring of pregnancy follow-up visits, may have influenced the time devoted to nutrition education.

## 6. Conclusions

Although most of the Spanish pregnant women surveyed showed good adherence to the Mediterranean pattern, there is room for improvement in their habits, as a reduced intake of foods of great nutritional interest, such as legumes, nuts, fish and seafood, was observed, as was a high consumption of other less recommendable foods, such as confectionery. In addition, a low level of physical activity has been found in this group. The information on food and nutrition received by Spanish pregnant women is mostly of a general nature and their knowledge on the subject is very limited. Despite this, pregnant women who followed the recommendations received and those who demonstrated greater knowledge on the subject had better habits, which shows the potential of nutrition education during pregnancy. Thus, personalized advice could be a key point to maximize the quality of the diet of pregnant women, which is so important at this stage of the woman’s life cycle. Future research should investigate why midwives opt for general and not very personalized advice in order to assess whether nutrition education of pregnant women should be carried out by a multidisciplinary team with professionals specifically trained in the field of food and nutrition.

## Figures and Tables

**Table 1:** Adherence to the Mediterranean diet pattern during pregnancy based on various sociodemographic variables and characteristics of the pregnant women.

	TOTAL (Burgos + Granada)							Burgos							Granada						
	TOTAL (N = 771)		LOW (N = 171)		HIGH (N = 600)		*p*-Value	TOTAL (N = 383)		LOW (N = 91)		HIGH (N = 292)		*p*-Value	TOTAL (N = 388)		LOW (N = 80)		HIGH (N = 308)		*p*-Value
	N	%	N	%	N	%		N	%	N	%	N	%		N	%	N	%	N	%	
Age (years)							<0.001							<0.001							0.008
<35	427	55.4	120	70.2	307	51.2		206	53.8	64	70.3	142	48.6		221	57.0	56	70.0	165	53.6	
>=35	344	44.6	51	29.8	293	48.8		177	46.2	27	29.7	150	51.4		167	43.0	24	30.0	143	46.4	
Country							0.022							0.055							0.354
Spain	693	90.0	146	85.4	547	91.3		326	85.3	72	79.1	254	87.3		367	94.6	74	92.5	293	95.1	
Others	77	10.0	25	14.6	52	8.7		56	14.7	19	20.9	37	12.7		21	5.4	6	7.5	15	4.9	
Education							0.012							0.634							0.002
University	303	39.3	53	31.0	250	41.7		147	38.4	33	36.3	114	39		156	40.2	20	25.0	136	44.2	
Non-University	468	60.7	118	69.0	350	58.3		236	61.6	58	63.7	178	61		232	59.8	60	75.0	172	55.8	
Prior illness							0.524							0.972							0.242
Yes	92	12.1	18	10.7	74	12.5		54	14.2	13	14.3	41	14.1		38	9.9	5	6.4	33	10.9	
No	671	87.9	151	89.3	520	87.5		327	85.8	78	85.7	249	85.9		344	90.1	73	93.6	271	89.1	
Previous pregnancy							0.397							0.725							0.485
None	381	49.8	88	52.1	293	49.2		176	46.7	43	48.3	133	46.2		205	52.8	45	56.3	160	51.9	
1	272	35.6	53	31.4	219	36.7		144	38.2	31	34.8	113	39.2		128	33.0	22	27.5	106	34.4	
2 or more	112	14.6	28	16.6	84	14.1		57	15.1	15	16.9	42	14.6		55	14.2	13	16.3	42	13.6	
Miscarriage							0.777							0.492							0.812
Yes	272	35.4	59	34.5	213	35.7		146	38.2	32	35.2	114	39.2		126	32.6	27	33.8	99	32.4	
No	496	64.6	112	65.5	384	64.3		236	61.8	59	64.8	177	60.8		260	67.4	53	66.3	207	67.6	
Pregnancy problems							0.813							0.318							0.478
Yes	144	18.8	31	18.1	113	18.9		63	16.6	12	13.2	51	17.6		81	20.9	19	23.8	62	20.1	
No	624	81.3	140	81.9	484	81.1		317	83.4	79	86.8	238	82.4		307	79.1	61	76.3	246	79.9	
Previous BMI							0.027							0.334							0.032
Underweight	35	4.7	6	3.7	29	5.0		17	4.7	2	2.3	15	5.4		18	4.8	4	5.3	14	4.7	
Normal weight	457	61.8	91	55.5	366	63.7		221	60.9	49	56.3	172	62.3		236	62.9	42	55.3	194	64.9	
Overweight	152	20.6	35	21.3	117	20.3		74	20.4	21	24.1	53	19.2		78	20.8	14	18.4	64	21.4	
Obesity	95	12.9	32	19.5	63	11.0		51	14	15	17.2	36	13		43	11.5	16	21.1	27	9.0	

The sum of the frequencies did not reach the value of 771 (sample size) when one of the responses was ‘don’t know or no answer’. ‘Low’ and ‘High’ refer to the level of adherence to the Mediterranean diet pattern. Cut off score for low and high adherence was < and ≥7 points in a modified MEDAS-14 questionnaire, respectively. The statistical test used is Chi-square.

**Table 2:** Adherence to the items of the MEDAS-14 questionnaire and level of physical activity during pregnancy.

	TOTAL (Burgos + Granada) (N = 771)				Burgos (N = 383)				Granada (N = 388)				
	Meet		Not Meet		Meet		Not Meet		Meet		Not Meet		
	N	%	N	%	N	%	N	%	N	%	N	%	*p*-Value
Olive Oil use	737	95.6	34	4.4	359	93.7	24	6.3	378	97.4	10	2.6	0.013
Olive oil	315	40.9	456	59.1	142	37.1	241	62.9	173	44.6	215	55.4	0.034
Vegetables	442	57.3	329	42.7	214	55.9	169	44.1	228	58.8	160	41.2	0.418
Fruits	388	50.3	383	49.7	213	55.6	170	44.4	175	45.1	213	54.9	0.004
Red meat	623	80.8	148	19.2	292	76.2	91	23.8	331	85.3	57	14.7	0.001
Butter, margarine or cream	706	91.6	65	8.4	357	93.2	26	6.8	349	89.9	39	10.1	0.103
Sweet beverages	690	89.5	81	10.5	356	93.0	27	7.0	334	86.1	54	13.9	0.002
Legumes	227	29.4	544	70.6	105	27.4	278	72.6	122	31.4	266	68.6	0.220
Fish	191	24.8	580	75.2	124	32.4	259	67.6	67	17.3	321	82.7	<0.001
Non-homemade pastries	444	57.3	331	42.7	198	51.7	185	48.3	246	63.4	146	37.6	0.002
Nuts	290	37.6	481	62.4	126	32.9	257	67.1	164	42.3	224	57.7	0.007
White meat preference	667	86.5	104	13.5	325	84.9	58	15.1	342	88.1	46	11.9	0.182
Sofrito	476	61.7	295	38.3	207	54.0	176	46.0	269	69.3	119	30.7	<0.001
Wine	710	92.1	61	7.9	349	91.1	34	8.9	361	93.0	27	7.0	0.324
Physical activity	353	45.9	416	54.1	174	45.7	207	54.3	179	46.1	209	53.9	0.897

“Meets/Does not meet” refers to pregnant women who adhere/do not adhere to the recommendations, respectively. The statistical test used is Chi-square.

**Table 3:** Changes in habits during pregnancy.

	TOTAL (Burgos + Granada)							Burgos							Granada						
	TOTAL (N = 771)		LOW (N = 171)		HIGH (N = 600)		*p*-Value	TOTAL (N = 383)		LOW (N = 91)		HIGH (N = 292)		*p*-Value	TOTAL (N = 388)		LOW (N = 80)		HIGH (N = 308)		*p*-Value
	N	%	N	%	N	%		N	%	N	%	N	%		N	%	N	%	N	%	
OLIVE OIL							0.809							0.815							0.836
Higher	20	2.6	4	2.4	16	2.7		12	3.2	3	3.4	9	3.1		8	2.1	1	1.3	7	2.3	
Lower	98	12.9	24	14.3	74	12.5		52	13.8	14	15.7	38	13.1		46	12.0	10	12.7	36	11.8	
As before	644	84.5	140	83.3	504	84.8		314	83.1	72	80.9	242	83.7		330	85.9	68	86.1	262	85.9	
VEGETABLES							0.001							0.058							0.012
Higher	250	32.5	41	24.1	209	34.9		133	34.9	24	26.7	109	37.5		117	30.2	17	21.3	100	32.5	
Lower	51	6.6	20	11.8	31	5.2		20	5.2	8	8.9	12	4.1		31	8.0	12	15.0	19	6.2	
As before	468	60.9	109	64.1	359	59.9		228	59.8	58	64.4	170	58.4		240	61.9	51	63.8	189	61.4	
FRUITS							0.101							0.861							0.035
Higher	374	48.8	76	45.0	298	49.9		201	52.6	46	50.5	155	53.3		173	45.1	30	38.5	143	46.7	
Lower	40	5.2	14	8.3	26	4.4		18	4.7	5	5.5	13	4.5		22	5.7	9	11.5	13	4.2	
As before	352	46.0	79	46.7	273	45.7		163	42.7	40	44.0	123	42.3		189	49.2	39	50.0	150	49.0	
RED MEAT							0.022							0.094							0.045
Higher	49	6.4	16	9.6	33	5.5		33	8.7	9	10.1	24	8.3		16	4.2	7	9.1	9	3.0	
Lower	313	41.1	55	33.1	258	43.4		148	39.1	26	29.2	122	42.1		165	43.2	29	37.7	136	44.6	
As before	399	52.4	95	57.2	304	51.1		198	52.2	54	60.7	144	49.7		201	52.6	41	53.2	160	52.5	
BUTTER/CREAM							0.616							0.431							0.176
Higher	38	5.1	8	4.8	30	5.2		16	4.4	6	6.8	10	3.6		22	5.9	2	2.5	20	6.7	
Lower	212	28.6	43	25.7	169	29.4		105	28.7	24	27.3	81	29.1		107	28.5	19	24.1	88	29.6	
As before	492	66.3	116	69.5	376	65.4		245	66.9	58	65.9	187	67.3		247	65.7	58	73.4	189	63.6	
SWEET BEVERAGES							0.355							0.151							0.942
Higher	39	5.3	12	7.2	27	4.7		16	4.4	7	8.0	9	3.3		23	6.1	5	6.4	18	6.0	
Lower	325	43.9	75	45.2	250	43.6		166	45.7	41	46.6	125	45.5		159	42.2	34	43.6	125	41.8	
As before	376	50.8	79	47.6	297	51.7		181	49.9	40	45.5	141	51.3		195	51.7	39	50.0	156	52.2	
LEGUMES							0.359							0.056							0.159
Higher	115	15.0	31	18.1	84	14.0		64	16.8	22	24.2	42	14.5		51	13.1	9	11.3	42	13.6	
Lower	62	8.1	15	8.8	47	7.9		29	7.6	4	4.4	25	8.6		33	8.5	11	13.8	22	7.1	
As before	592	77.0	125	73.1	467	78.1		288	75.6	65	71.4	223	76.9		304	78.4	60	75.0	244	79.2	
FISH/SEAFOODS							0.539							0.678							0.136
Higher	99	12.9	24	14.1	75	12.6		55	14.5	15	16.5	40	13.8		44	11.4	9	11.4	35	11.4	
Lower	93	12.1	24	14.1	69	11.6		41	10.8	8	8.8	33	11.4		52	13.5	16	20.3	36	11.7	
As before	574	74.9	122	71.8	452	75.8		284	74.7	68	74.7	216	74.7		290	75.1	54	68.4	236	76.9	
NON-HOMEMADE PATRIES							0.005							0.049							0.073
Higher	180	23.7	53	31.2	127	21.5		89	23.5	27	29.7	62	21.5		91	23.9	26	32.9	65	21.6	
Lower	163	21.4	24	14.1	139	23.6		82	21.6	12	13.2	70	24.3		81	21.3	12	15.2	69	22.9	
As before	417	54.9	93	54.7	324	54.9		208	54.9	52	57.1	156	54.2		208	54.7	41	51.9	167	55.5	
NUTS							0.005							0.048							0.034
Higher	175	23.1	25	14.7	150	25.5		80	21.3	11	12.1	69	24.2		95	24.8	14	17.7	81	26.6	
Lower	73	9.6	23	13.5	50	8.5		36	9.6	10	11.0	26	9.1		37	9.7	13	16.5	24	7.9	
As before	511	67.3	122	71.8	389	66.0		260	69.1	70	76.9	190	66.7		251	65.5	52	65.8	199	65.5	
WHITE MEAT							0.768							0.881							0.851
Includes	62	8.1	15	8.8	47	7.9		34	8.9	9	9.9	25	8.6		28	7.3	6	7.7	22	7.2	
Excludes	14	1.8	4	2.4	10	1.7		7	1.8	2	2.2	5	1.7		7	1.8	2	2.6	5	1.6	
As before	692	90.1	151	88.8	541	90.5		342	89.3	80	87.9	262	89.7		348	90.9	70	89.7	278	91.1	
SOFRITO							0.107							0.295							0.101
Higher	39	5.1	12	7.1	27	4.5		18	4.7	4	4.4	14	4.8		21	5.5	8	10.1	13	4.2	
Lower	120	15.7	33	19.4	87	14.6		67	17.6	21	23.1	46	15.9		53	13.8	12	15.2	41	13.4	
As before	606	79.2	125	73.5	481	80.8		295	77.6	66	72.5	229	79.2		311	80.8	59	74.7	252	82.4	
WINE							0.343							0.152							0.040
Higher	2	0.3	1	0.7	1	0.2		1	0.3	1	1.2	0	0.0		1	0.3	0	0.0	1	0.4	
Lower	338	48.6	68	44.4	270	49.7		171	49.7	44	53.0	127	48.7		167	47.4	24	34.3	143	50.7	
As before	356	51.1	84	54.9	272	50.1		172	50.0	38	45.8	134	51.3		184	52.3	46	65.7	138	48.9	
PHYSICAL ACTIVITY							0.457							0.163							0.803
More activity	144	18.9	28	16.6	116	19.5		73	19.3	15	16.5	58	20.1		71	18.5	13	16.7	58	19.0	
Less activity	297	38.9	63	37.3	234	39.4		151	39.8	31	34.1	120	41.7		146	38.0	32	41.0	114	37.3	
Similar activity	322	42.2	78	46.2	244	41.1		155	40.9	45	49.5	110	38.2		167	43.5	33	42.3	134	43.8	

The sum of the frequencies did not reach the sample size of 771 when some responses were ‘don’t know or no answer’. ‘Low’ and ‘High’ refer to the level of adherence to the Mediterranean diet pattern. Cut off score for low and high adherence was < and ≥7 points in a modified MEDAS-14 questionnaire, respectively. The statistical test used is Chi-square.

**Table 4:** Knowledge about nutrition in pregnant women.

	TOTAL (N = 771)		Burgos (N = 383)		Granada (N = 388)		
SINGLE-ANSWER QUESTIONS	N	%	N	%	N	%	*p*-Value
*Are there any differences in energy requirements during the different trimesters of pregnancy?*							0.102
Yes	584	89.0	281	87.0	303	91.0	
No	72	11.0	42	13.0	30	9.0	
*What is the recommended weight gain for a woman who started her pregnancy at a normal weight (BMI = 18.5–24.9 kg/m^2^)?*							0.388
9.0–12.0 kg	474	76.0	239	78.1	235	73.9	
11.5–16.0 kg	137	22.0	60	19.6	77	24.2	
17.0–22.5 kg	9	1.4	4	1.3	5	1.6	
23.0–28.5 kg	4	0.6	3	1.0	1	0.3	
*When should folic acid supplements be taken to prevent neural tube defects?*							0.300
During the first trimester of pregnancy	26	3.7	8	2.3	18	4.9	
When she first knows that she is pregnant	100	14.1	50	14.6	50	13.6	
During the whole pregnancy	350	49.3	174	50.7	176	48.0	
At least one month before pregnancy and during the first three months of pregnancy	234	33.0	111	32.4	123	33.5	
*What are the recommended daily servings of dairy to meet calcium requirements during pregnancy?*							0.001
1–2	215	33.1	91	26.9	124	39.7	
3–4	415	63.8	232	68.6	183	58.7	
5 or more	20	3.1	15	4.4	5	1.6	
**MULTIPLE-ANSWER QUESTIONS**							
*Among the following foods, which ones do you consider to be rich in iron?*							0.859
Red meat	364	47.7	181	47.5	183	48.0	
Legumes	660	86.5	341	89.5	319	83.7	
Fish	159	20.8	84	22.0	75	19.7	
Green leafy vegetables	382	50.1	186	48.8	196	51.4	
All correct selected	56	7.3	29	7.6	27	7.1	
*Among the following foods, which ones do you consider to have the most bioavailable iron?*							0.248
Red meat	211	27.7	109	28.6	102	26.8	
Legumes	395	51.8	201	52.8	194	50.9	
Fish	69	9.0	34	8.9	35	9.2	
Green leafy vegetables	207	27.1	89	23.4	118	31.0	
All correct selected	24	3.1	9	2.4	15	3.9	
*Among the following proposals, which can help combat constipation during pregnancy?*							0.449
Adequate water intake	629	82.4	321	84.3	308	80.8	
Physical activity	564	73.9	278	73.0	286	75.1	
Fruits and vegetables	653	85.6	315	82.7	338	88.7	
Meat	9	1.2	2	0.5	7	1.8	
All correct selected	474	62.1	233	61.2	241	63.3	
*Among the following options, which can help reduce nausea and vomiting during pregnancy?*							0.142
Drinking plenty of fluids during meals	158	20.7	79	20.7	79	20.7	
Avoid fatty or spicy foods	519	68.0	261	68.5	258	67.7	
Reduce the number of meals per day	85	11.1	41	10.8	44	11.5	
Minimize odors while cooking	119	15.6	70	18.4	49	12.9	
Snacking	111	14.5	45	11.8	66	17.3	
Eat something solid before getting up in the morning	304	39.8	159	41.7	145	38.1	
All correct selected	33	4.3	20	5.2	13	3.4	
*Which fish should be avoided during pregnancy because of high mercury levels?*							<0.001
Swordfish	509	66.7	254	66.7	255	66.9	
Anchovies	88	11.5	27	7.1	61	16.0	
Tuna	420	55.0	203	53.3	217	57.0	
Pike	75	9.8	47	12.3	28	7.3	
Bluefin tuna	495	64.9	252	66.1	243	63.8	
Shark	420	55.0	215	56.4	205	53.8	
Horse mackerel	35	4.6	22	5.8	13	3.4	
Salmon	104	13.6	35	9.2	69	18.1	
All correct selected	15	2.0	12	3.1	3	0.8	
*Which foods should be avoided during pregnancy because of listeria/toxoplasma-related risks?*							0.405
Salads in a bag	177	23.2	98	25.7	79	20.7	
Cured raw meat products (chorizo, sausage, cured ham…)	716	93.8	354	92.9	362	95.0	
Refrigerated pate (not sterilized)	607	79.6	294	77.2	313	82.2	
Unpasteurized milk and milk products	626	82.0	311	81.6	315	82.7	
Smoked fishery products requiring refrigeration	475	62.3	233	61.2	242	63.5	
Raw fish or shellfish	652	85.5	317	83.2	335	87.9	
All correct selected	140	18.3	80	21.0	60	15.7	

The sum of the frequencies did not reach the sample size of 771 when some responses were ‘don’t know or no answer’. The answer that appears underlined is the correct one. The statistical test used is Chi-square.

**Table 5:** Pregnant women’s perception of various dietary issues based on their adherence to the Mediterranean diet.

	TOTAL (Burgos + Granada)							Burgos							Granada						
QUESTIONS	TOTAL (N = 771)		LOW (N = 171)		HIGH (N = 600)		*p*-Value	TOTAL (N = 383)		LOW (N = 91)		HIGH (N = 292)		*p*-Value	TOTAL (N = 388)		LOW (N = 80)		HIGH (N = 308)		*p*-Value
	N	%	N	%	N	%		N	%	N	%	N	%		N	%	N	%	N	%	
Do you think inadequate weight gain during pregnancy can pose a risk to the health of the mother and the baby?							0.004							0.027							0.136
Low risk	30	3.9	10	5.9	20	3.4		15	4.0	6	6.7	9	3.1		15	3.9	4	5.0	11	3.6	
Moderate risk	74	9.6	26	15.3	48	8.0		37	9.8	14	15.6	23	8.0		37	9.5	12	15.0	25	8.1	
High risk	663	86.4	134	78.8	529	88.6		327	86.3	70	77.8	257	88.9		336	86.6	64	80.0	272	88.3	
How important do you think is nutrition during pregnancy							<0.001							0.191							<0.001
Not important	5	0.7	4	2.4	1	0.2		1	0.3	0	0.0	1	0.3		4	1.0	4	5.1	0	0.0	
Moderately important	28	3.7	13	7.6	15	2.5		20	5.2	8	8.8	12	4.1		8	2.1	5	6.3	3	1.0	
Very important	734	95.7	153	90.0	581	97.3		360	94.5	83	91.2	277	95.5		374	96.9	70	88.6	304	99.0	
Do you believe that your diet during pregnancy can affect the health of the baby?							0.002							0.042							0.054
Little impact	18	2.3	6	3.5	12	2.0		11	2.9	4	4.4	7	2.4		7	1.8	2	2.5	5	1.6	
Moderate impact	30	3.9	14	8.2	16	2.7		20	5.2	9	9.9	11	3.8		10	2.6	5	6.3	5	1.6	
Significant impact	719	93.7	150	88.2	569	95.3		350	91.9	78	85.7	272	93.8		369	95.6	72	91.1	297	96.7	

The sum of the frequencies did not reach the sample size of 771 when some responses were ‘don’t know or no answer’. ‘Low’ and ‘High’ refer to the level of adherence to the Mediterranean diet pattern. Cutoff score for low and high adherence was < and ≥7 points in a modified MEDAS-14 questionnaire, respectively. The statistical test used is Chi-square.

**Table 6:** Nutritional education received at the midwife consultation and its relation to adherence to the Mediterranean diet.

	TOTAL (Burgos + Granada)							Burgos							Granada						
QUESTIONS	TOTAL (N = 771)		LOW (N = 171)		HIGH (N = 600)		*p*-Value	TOTAL (N = 383)		LOW (N = 91)		HIGH (N = 292)		*p*-Value	TOTAL (N = 388)		LOW (N = 80)		HIGH (N = 308)		*p*-Value
	N	%	N	%	N	%		N	%	N	%	N	%		N	%	N	%	N	%	
Did you receive written material on nutrition topics?							0.528							0.641							0.416
Yes	520	68.1	113	66.1	407	68.6		303	79.7	71	78.0	232	80.3		217	56.5	42	52.5	175	57.6	
No	244	31.9	58	33.9	186	31.4		77	20.3	20	22.0	57	19.7		167	43.5	38	47.5	129	42.4	
Did you receive oral information on nutrition topics?							0.780							0.372							0.572
Yes	610	79.9	138	80.7	472	79.7		323	85.0	80	87.9	243	84.1		287	74.9	58	72.5	229	75.6	
No	153	20.1	33	19.3	120	20.3		57	15.0	11	12.1	46	15.9		96	25.1	22	27.5	74	24.4	
Indicate the type of information received							0.938							0.716							0.783
General information	527	84.2	119	84.4	408	84.1		271	83.9	69	85.2	202	83.5		256	84.5	50	83.3	206	84.8	
Personalized information	99	15.8	22	15.6	77	15.9		52	16.1	12	14.8	40	16.5		47	15.5	10	16.7	37	15.2	
Assess the amount of information received							0.541							0.924							0.604
Little	157	24.6	31	21.8	126	25.4		69	20.9	16	19.5	53	21.4		88	28.5	15	25.0	73	29.3	
Moderate	231	36.2	50	35.2	181	36.4		117	35.5	29	35.4	88	35.5		114	36.9	21	35.0	93	37.3	
Significant	251	39.3	61	43.0	190	38.2		144	43.6	37	45.1	107	43.1		107	34.6	24	40.0	83	33.3	
Indicate if you have implemented the recommendations received							<0.001							0.001							0.218
Little	54	8.3	20	13.5	34	6.7		24	7.2	11	13.3	13			30	9.4	9	13.8	21	8.2	
Moderate	142	21.7	43	29.1	99	19.6		75	22.5	27	32.5	48	19.1		67	20.9	16	24.6	51	20.0	
Significant	458	70.0	85	57.4	373	73.7		235	70.4	45	54.2	190	75.7		223	69.7	40	61.5	183	71.8	
Evaluate your satisfaction with the information received							0.287							0.402							0.663
Little	132	20.1	33	22.0	99	19.5		52	15.6	15	18.1	37	14.7		80	24.8	18	26.9	62	24.3	
Moderate	182	27.7	34	22.7	148	29.2		95	28.4	19	22.9	76	30.3		86	26.7	15	22.4	71	27.8	
Significant	343	52.2	83	55.3	260	51.3		187	56.0	49	59.0	138	55.0		156	48.4	34	50.7	122	47.8	
Assess the increase in your knowledge during pregnancy							0.022							0.031							0.521
Little	182	23.7	42	24.7	140	23.5		91	23.9	23	25.3	68	23.4		91	23.6	19	24.1	72	23.5	
Moderate	205	26.7	58	34.1	147	24.6		110	28.9	35	38.5	75	25.9		95	24.6	23	29.1	72	23.5	
Significant	380	49.5	70	41.2	310	51.9		180	47.2	33	36.3	147	50.7		200	51.8	37	46.8	163	53.1	

The sum of the frequencies did not reach the sample size of 771 when some responses are ‘don’t know or no answer’. ‘Low’ and ‘High’ refer to the level of adherence to the Mediterranean diet pattern. Cutoff score for low and high adherence was < and ≥7 points in a modified MEDAS-14 questionnaire, respectively. The statistical test used is Chi-square.

## Data Availability

The original contributions presented in the study are included in the article, further inquiries can be directed to the corresponding author.
